# Novel Donepezil–Arylsulfonamide Hybrids as Multitarget-Directed Ligands for Potential Treatment of Alzheimer’s Disease

**DOI:** 10.3390/molecules26061658

**Published:** 2021-03-16

**Authors:** Fausto Queda, Sonia Calò, Karolina Gwizdala, João D. Magalhães, Sandra M. Cardoso, Sílvia Chaves, Luca Piemontese, M. Amélia Santos

**Affiliations:** 1 Centro de Química Estrutural and Departamento de Engenharia Química, Instituto Superior Técnico, Universidade de Lisboa, Av. Rovisco Pais, 1049-001 Lisboa, Portugal; fausto.queda@gmail.com (F.Q.); s.calo19@studenti.uniba.it (S.C.); karo.gwizdala@gmail.com (K.G.); silvia.chaves@tecnico.ulisboa.pt (S.C.); 2 Dipartimento di Farmacia–Scienze del Farmaco, Università degli Studi di Bari “Aldo Moro”, Via E. Orabona 4, I-70125 Bari, Italy; luca.piemontese@uniba.it; 3 CNC–Center for Neuroscience and Cell Biology, University of Coimbra, 3004-504 Coimbra, Portugal; joaoduartemagalhaes7@gmail.com (J.D.M.); cardoso.sandra.m@gmail.com (S.M.C.); 4 Institute of Molecular and Cell Biology, Faculty of Medicine, University of Coimbra, 3000-548 Coimbra, Portugal; 5 National Research Council (CNR)–ISPA, via Amendola 122/O, 70126 Bari, Italy

**Keywords:** arylsulfonamide, donepezil, anti-neurodegeneratives, Alzheimer´s disease, AChE inhibitors, A*β* aggregation

## Abstract

Alzheimer’s disease (AD) is one of the most devastating neurodegenerative disorders, characterized by multiple pathological features. Therefore, multi-target drug discovery has been one of the most active fields searching for new effective anti-AD therapies. Herein, a series of hybrid compounds are reported which were designed and developed by combining an aryl-sulfonamide function with a benzyl-piperidine moiety, the pharmacophore of donepezil (a current anti-AD acetylcholinesterase AChE inhibitor drug) or its benzyl-piperazine analogue. The in vitro results indicate that some of these hybrids achieve optimized activity towards two main AD targets, by displaying excellent AChE inhibitory potencies, as well as the capability to prevent amyloid-*β* (A*β*) aggregation. Some of these hybrids also prevented A*β*-induced cell toxicity. Significantly, drug-like properties were predicted, including for blood-brain permeability. Compound **9** emerged as a promising multi-target lead compound (AChE inhibition (IC_50_ 1.6 μM); A*β* aggregation inhibition 60.7%). Overall, this family of hybrids is worthy of further exploration, due to the wide biological activity of sulfonamides.

## 1. Introduction

Alzheimer’s disease (AD) is one of the most common devastating age-dependent neurodegenerative disorders of the central nervous system (CNS), characterized by a progressive neuronal degeneration associated to the loss of cognitive function. Multiple etiological hypotheses have been proposed for the onset and progression of AD, including genetics, oxidative stress, inflammatory–immunologic dysregulation and neurotransmitter dysfunctions. Notwithstanding the enormous worldwide research efforts aimed to elucidate AD pathology and to find an effective therapy, its multifaceted nature and the still not fully understood complexity of this disease are certainly the main reasons for the high rate of failure in drug development programs and the so far consequent absence of cure [1]. There are a few drugs approved by the US Food and Drug Administration (FDA) for the treatment of AD, but they can only provide some amelioration of memory deficits. Among them, four are acetylcholinesterase (AChE) inhibitors (*tacrine*, *donepezil*, *rivastigmine* and *galantamine),* to compensate for the loss of acetylcholine and one (*memantine)* is a N-methyl-D-aspartate receptor (NMDAR) antagonist, to regulate the excitatory glutamatergic function [2,3]. Excluding tacrine, which was withdrawn from the market due to hepatotoxicity, the other mentioned drugs are under current use, as well as the quite recently (in 2014) FDA approved donepezil-memantine combination drug (Namzaric), used in fixed doses. However, these drugs are not able to stop or reverse the progression of neurodegeneration, but only provide a short-term symptomatic relief in prodromal or early stages of AD [1,2,3]. Therefore, the identification of valid disease-modifying treatments for AD represents nowadays a very urgent and still unmet medical global problem [4,5].

The existence of diverse multiple factors, that can contribute to the onset and progression of AD, has been considered one of the main obstacles for drug development. However, several pathological hallmarks have been widely recognized, including the protein misfolding associated to amyloid cascade, with formation of amyloid aggregates (amyloid plaques) and tau hyperphosphorylation; the collapse of the neurotransmitters (cholinergic dysfunction), oxidative stress and metal dyshomeosthasis [6]. Thus, over the years, the development of a multi-target therapeutics [7] has become an important approach widely used in the research of new drugs, envisaging the simultaneous interaction with several pathological targets. Among the different drug development strategies, many hybrid molecules have been explored by combining in the same molecular entity approved anti-AD drugs, or their determinant pharmacophores, with other biologically active molecular moieties to enable the hitting of other important AD targets, such as amyloid beta (A*β*) aggregates, radical oxygen species (ROS), redox-active metal ions and enzymes (e.g., monoamine oxidase, beta-secretase 1) [8,9,10,11,12,13].

Following the conceptualization of the multi-target strategy for anti-AD drugs and the associated interest of this research group on the repositioning of approved drugs [14,15,16], the present work is focused on the development of new arylsulfonamide-donepezil like molecules with potential ability to interact with different targets of AD. The option for aromatic sulfonamides, as second pharmacophore, is due to their wide clinical applications, namely as antibacterial, antifungal, anti-cancer and anti-depressant [17]. Furthermore, a number of sulfonamide-containing hybrids, enclosing tacrine/quinoline moieties, have been recently developed as promising anti-AD drugs, exhibiting good capacity for the inhibition of cholinesterases and self-induced A*β* aggregation, as well as antioxidant activity [18,19,20].

**Scheme 1:**
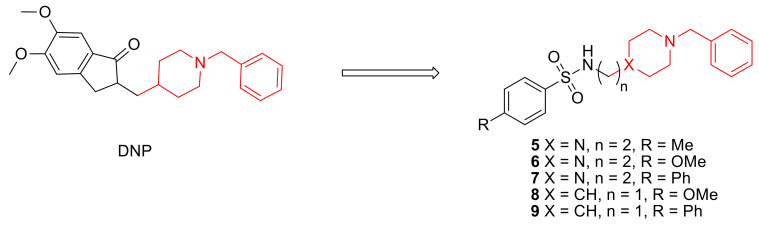
Donepezil (DNP) and the studied DNP-like hybrids.

In particular, we present herein the design, synthesis and biological evaluation of a small library of donepezil-sulfonamides, which are based on the attachment of a DNP active pharmacophore moiety (benzylpiperidine/benzylpiperazine) to a set of *para*-substituted aromatic sulfonamides (see Scheme 1). In addition to the molecular design and synthesis, these novel compounds are biologically evaluated in vitro for inhibition of AChE and A*β* aggregation and then bio-assayed in cell environment for the neuroprotection properties. The new compounds are also evaluated for their pharmacokinetic properties and drug likeness. Discussion on some structural-activity relationships is also provided, namely effects of *para*-substituents modification and linker size on the biological activity of the hybrids.

## 2. Results and Discussion

### 2.1. Molecular Design and Docking

Since donepezil (DNP) is a molecule of high interest in the medical treatment of AD [2,3], its active pharmacophore was selected for the design of several new DNP-like hybrid molecules enclosing an aryl sulfonamide moiety. In particular, the rationale for these new hybrids was based on using, as the first pharmacophores, two donepezil-mimetic derivatives, namely the benzyl-piperidine moiety (**8** and **9**) (the active moiety of Donepezil, as AChEi) and its isostere benzyl-piperazine (**5**–**7**), to interact with the catalytic anionic site (CAS) of AChE. As secondary pharmacophores, the aryl sulfonamides were supposed to increase the AChE binding affinity, by mimicking the role of the indanone moiety of DNP on its extra-interaction with the peripheral active site (PAS) of AChE. In addition, enabling that potential dual mode of interaction with AChE, the aryl sulfonamides could also inhibit A*β* aggregation. Furthermore, sulfonamides are well-known moieties present in many approved drugs due to their wide biological activity [21].

**Figure 1:**
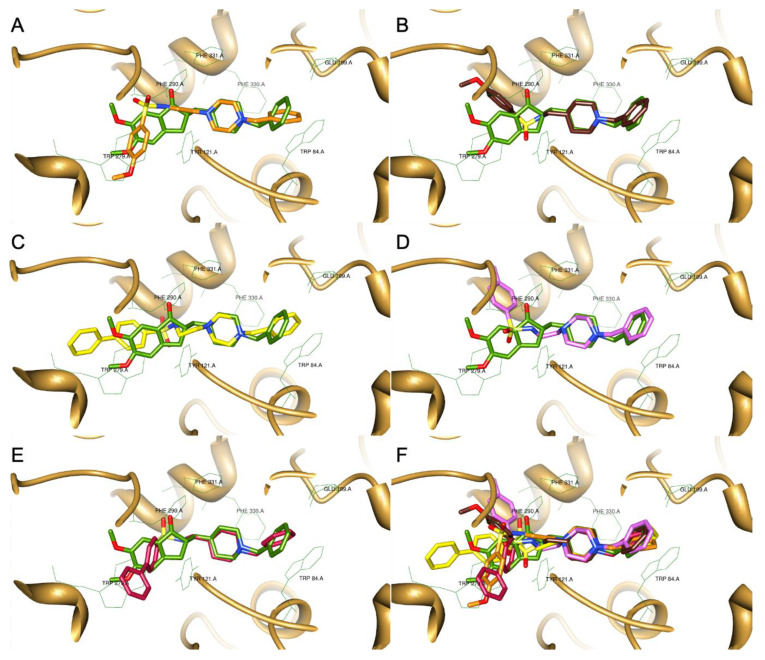
Superimposition of the different DNP-sulfonamide compounds with the original ligand (DNP-green), as a result of their docking inside the AChE active site (PDB code: 1EVE). (**A**) compound **6**; (**B**) compound **8**; (**C**) compound **7**; (**D**) compound **5;** (**E**) compound **9**; (**F**) all the compounds.

Modelling docking studies of this set of new compounds into AChE were performed in order to get some insight on their binding interactions with the enzyme active site and to understand potential structure-activity relationships. Firstly, from an X-ray structure of a DNP-AChE complex, the co-crystallized ligand (DNP) was docked back into the AChE binding site of the enzyme and showed good superimposition with the native ligand. Being satisfied with the good overlay of the simulated and original co-crystallized DNP, the same docking protocol was used to dock the target compounds (**5**–**9**). The best docked poses of all the synthesized compounds were selected and superimposed with that of DNP, as shown in Figure 1A–E. Analysis of the docking graphics shows that all the compounds have the pharmacophore benzyl-piperazinium (**5**–**7**) and benzyl-piperidinium moieties (**8** and **9**) anchored to the CAS of AChE (bottom of the gorge), through binding interactions via aromatic π-π stacking with the phenyl ring from Trp84 (A). Among this set of compounds, those bearing a benzyl-piperidine group (**8** (B) and **9** (E)), showed the best superimposition with the same moiety of DNP, while some other compounds (**6** (A), **7** (C)) present a little bending of the benzyl group relatively to that of DNP. At the middle of the gorge, the charged nitrogen atom can establish a cation–π binding interaction with the phenyl group of Phe330. Compound **9** (E) shows a further superimposition between the sulfonyl group of the biphenyl-sulfonamide moiety and the carbonyl group of the DNP indanone. Both these groups, in DNP and **9** (E), can similarly interact with Phe290, but for some compounds, as **8** (B), this is not favored because they are pointed away from each other. These differences are reflected in the orientation of the aryl-sulfonyl groups, which in some compounds, as **5** (D)**,** appears distorted from that of the DNP indanone, possibly allowing positive interactions with other residues. For the biphenyl derivatives (**7** (C) and **9** (E)), the corresponding biaryl-sulfonyl groups seem to adopt a spatial orientation similar to that of the indanone moiety of DNP and so a similar π–π stacking interaction with the peripheral anionic residue, namely Trp279, is kept.

#### Pharmacokinetic Properties

Pharmacokinetic properties were calculated with the software QikProp v.2.5 (Schrödinger, LLC, New York, NY, USA) [22] and they are depicted in Table 1.

All the developed compounds are free from violations of Lipinski’s rule, thus, predicting their drug-likeness as oral active anti-AD agents and so they can be potentially considered for oral formulation [23]. The molecular weight (under 500) and the lipophilic character (*c*log *P* = 1.9–4.7) are two important parameters that allow anticipation of a good absorption and bioavailability [24]. Log BB was calculated to evaluate the capacity of the compounds to cross the blood-brain barrier (BBB) and the values obtained are also within the range calculated for drugs (−3 to 1) [22]. Caco permeability indicates the absorption from the intestine to the bloodstream. Considering that values lower than 25 nm/s and higher than 500 nm/s represent, respectively, a poor and a great absorption, it can be inferred that compounds **8** (363 nm/s) and **9** (457 nm/s) present a good Caco permeability (gut absorption), though lower than the drug DNP (893 nm/s), while **5**, **6** and **7** present a more moderate one.

Overall, these predictions indicate that this set of DNP derivatives presents good drug-like pharmacokinetic properties, in particular compounds **8** and **9** with good BBB permeability (log BB) and activity in CNS (+) as the parent drug, suggesting they can be good therapeutic candidates, even for oral administration.

### 2.2. Synthesis

**Scheme 2:**
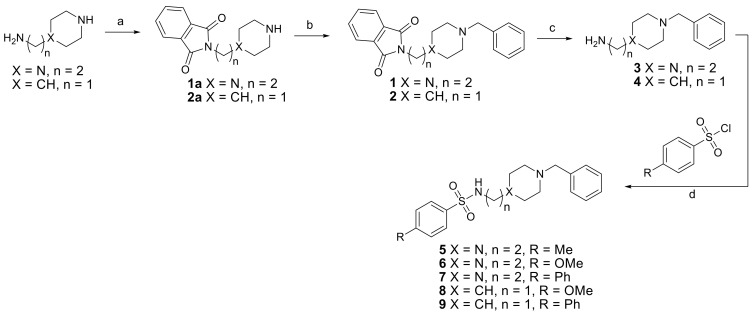
Synthetic plan for compounds **5**–**9**. Reagents and conditions: (**a**) Phthalic anhydride 160–180 °C, 4 h; (**b**) BnBr, KOH, EtOH, rt, 24 h to obtain **1** or BnBr, TEA, K_2_CO_3_, ACN, 50 °C, 3 h to obtain **2**; (**c**) MeNH_2_ (40% aqueous solution) rt, 24 h; (**d**) TEA, dioxane, rt, 12 h.

Five hybrid compounds were obtained by conjugation of two DNP mimetic moieties, namely the *N*-benzylated piperidine and piperazine derivatives, with *para*-substituted arylsulfonamides, following the synthetic procedure depicted in Scheme 2.

For the preparation of the target hybrids, two starting compounds were selected, namely piperidine and piperazine derivatives bearing alkylamine groups (**1a** and **2a**), for the subsequent attachment to the arylsulfonyl moieties. So, the first objective was to introduce a permanent benzyl group at the secondary amine of the piperidine and piperazine rings. However, the success of this alkylation required a previous selective temporary protection of the primary amines of the alkyl amine side groups. Therefore, a phthalimide group was chosen as protecting group because of its “orthogonality” relative to the benzyl group. Thus, the preparation of compounds **1** and **2** relied on the phthalamidation of the bearing primary amines of the corresponding precursors (**1a** and **2a**) by its reaction with phthalic anhydride at 160–180 °C for 4 h, under neat conditions (Scheme 2, step a), according to previously reported [14,25].

For the subsequent benzylation of the secondary cyclic amines, benzyl bromide (BnBr) was used with different solvent and base conditions (Scheme 2, step b), mainly due to the poor solubility of the piperidine derivative in EtOH. Nevertheless, the reactions went on smoothly, with 50% yield for **1** and 29% yield for **2,** after two steps. The subsequent step towards the preparation of compounds **3** and **4** consisted in the removal of the phthalimide group, to leave the primary amine free for the next reaction (coupling of the two main moieties). To perform this, a well reported procedure was carried out, using MeNH_2_ in 40% aqueous solution, to afford **3** and **4** in 97% and 69% yield, respectively (Scheme 2, step c) [26]. The last reaction (Scheme 2, step d) involved the coupling of the free amine intermediates (**3, 4**) with the different commercially available aryl-sulfonyl chlorides, via sulfonamide formation. This reaction was carried out in dioxane and the final pure compounds (**5**–**9**) were obtained in moderate yields (30–49%).

### 2.3. Biological Assays in Solution

#### 2.3.1. Acetylcholinesterase Inhibition

In Table 2 the biological activity results for the synthetized compounds (**5**–**9**) are summarized and compared with two well-known standard compounds, namely in terms of AChE inhibition and inhibition of self-induced A*β* aggregation.

Compounds **5** and **6** do not show significant inhibition of AChE, which can be related with the lower affinity of the piperazine ring, as compared with the piperidinic one, for the AChE binding site and also to the higher linker size (n = 2) of these compounds when compared with that of derivatives **8** and **9** (n = 1). As already shown in Figure 1, hybrids with higher length linker presented some bending, relatively to DNP, in the CAS of AChE. The fact that the inhibitory activity of **7**, also containing a piperazine moiety and n = 2, is higher than that of compounds **5** and **6** should be attributed to its biphenyl group. Moreover, compounds **8** and **9** presented higher AChE inhibition (IC_50_ 1.6–6.2 μM) than the compounds with piperazine moiety (38.7 < IC_50_ > 100 μM) and again the biphenyl compound (**9**) is the most potent, which is in accordance with the docking studies (see Section 2.1). The better inhibitory activity of the biphenyl derivatives can be explained in two ways: the phenyl substituent of these inhibitors can promote extra binding interactions inside the active site of the enzyme, namely with the aromatic residues of PAS, as suggested by the inhibitor-enzyme docking simulations; and, the long size of this group can force some structural rearrangements of the inhibitors and promote some additional hydrophobic interaction between the inhibitor and the active site, thus further strengthening the inhibition.

Although the hybrids studied herein are poorer AChE inhibitors than DNP (IC_50_ = 0.026 μM [3]), the proposed series of compounds is still a valid research topic focused on a multi-target approach for anti-AD drug.

#### 2.3.2. Inhibition of A*β*_1–42_ Aggregation

Both drugs, tacrine and DNP, have moderate/weak capacity for the inhibition of A*β*_1–42_ aggregation, in opposition to their good activity as AChE inhibitors, as already mentioned in Section 2.3.1. Although all compounds (**5**–**9**) show moderate to good results in this assay, compounds **8** and **9,** enclosing the benzylpiperidine moiety, are better inhibitors of A*β* aggregation than tacrine, which is by far superior to DNP and are the strongest inhibitors among the hybrids under study. Therefore, the hybrids enclosing the benzylpiperidine moiety and a shorter linker size (n = 1) show better A*β* aggregation inhibition when compared to those containing a benzylpiperazine unit and n = 2. Moreover, for each type of derivative (benzylpiperidine/benzylpiperazine), it is possible to observe the same sequence of inhibitory capacity depending on the sulfonamide substitute (methoxy- > biphenyl-). Furthermore, among the benzylpiperazine derivatives, compound **5** with the methyl-arylsulfonamide group seems to be the best one.

The observed inhibitory ability dependence on some ligand structural features may be mainly explained by the different capacity for intercalation of the compounds into the fibrils that seems to be more difficult for the benzylpiperazine derivatives herein developed.

### 2.4. Cell Viability and Neuroprotection

**Figure 2:**
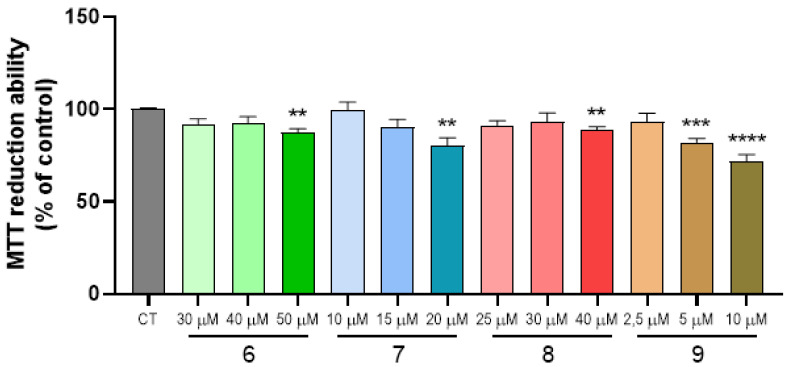
Dose-response screening of compounds **6–9** on SH-SY5Y neuronal cell line. SH-SY5Y cells were treated with varying concentrations of compounds from 2.5 μM to 50 μM for 24 h and cellular viability was measured by using the MTT reduction assay. Results are expressed relatively to SH-SY5Y untreated cells, with the mean ± SEM derived from at least three different experiments. ** *p* < 0.01, *** *p* < 0.001, **** *p* < 0.0001 significantly different when compared with control, untreated SH-SY5Y cells.

**Figure 3:**
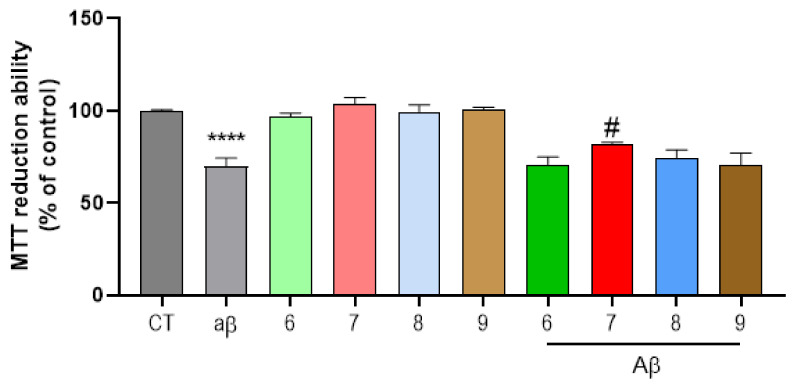
Neuroprotective effect of substances on A*β*_42_-induced toxicity on SH-SY5Y cells. SH-SY5Y cells were treated with A*β*_42_ peptide (2.5 μM) for 24 h in the absence or in the presence of the compounds. When compounds were present, a pre-incubation of 1 h preceded the A*β*_42_ treatment. Cellular viability was measured by using the MTT reduction assay. Results are expressed relatively to SH-SY5Y untreated cells, with the mean ± SEM derived from at least 3 different experiments. **** *p* < 0.0001, significantly different when compared with SH-SY5Y untreated cells (CT); # *p* < 0.05, significantly different when compared with A*β*_42_ treated SH-SY5Y cells. Compounds: **6** (40 µM); **7** (15) µM; **8** (30 µM); **9** (2.5 µM).

The approved therapy for AD is still scarce and ineffective. Thus, the need for new drugs that will help treat AD patients is essential. As such, we performed a dose-response screening to determine the highest non-toxic concentration of several hybrid compounds (Figure 2). Deposit of amyloid plaques is the most typical histopathological feature of AD. These extracellular agglomerates are typically constituted by fibrils of oligomerized A*β*_1–42_ [28]. Hence, we sought to challenge SH-SY5Y cells with A*β*_1–42_ peptides, as an in vitro model, for this study. As observed in Figure 3, A*β*_1–42_ exposure for 24 h was enough to decrease cell viability. Giving that A*β* deposition is deleterious to diseased patients, our results demonstrate that compound **7** was able to relapse the neurotoxicity induced by A*β* peptides in our cells (Figure 3) while compounds **8** and **9** showed more modest results in reverting neurotoxicity provoked by A*β*. Therefore, this kind of compounds deserves further studies, since they can be seen as suitable candidates to impede A*β* oligomerization and further toxic effects.

**Figure 4:**
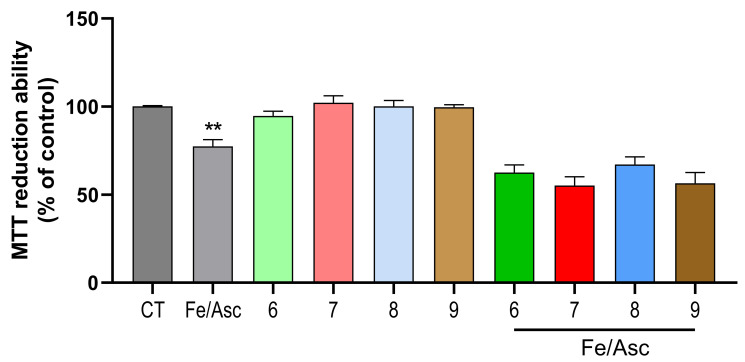
Neuroprotective effect of compounds on Ferrous Sulfate (Fe)/L-Ascorbic Acid (AscH(−))-induced toxicity on SH-SY5Y cells. Overnight cultures were treated with Fe/Asc (2.5 M/5 mM, respectively) for 24 h in the absence or in the presence of the compounds. When compounds were present, a pre-incubation of 1 h preceded the A*β*_42_ treatment. Cellular viability was determined by employing the MTT reduction assay. Results are expressed relatively to SH-SY5Y untreated cells, with the mean ± SEM derived from at least 3 different experiments. ** *p* < 0.01, significantly different when compared with SH-SY5Y untreated cells (CT). Compounds: **6** (40 µM); **7** (15 µM); **8** (30 µM); **9** (2.5 µM).

A*β* plaques formation is an intricate process, yet to be fully discovered. However, gathered data suggests that the production of reactive oxygen species (ROS) has a prominent role in this event [29]. For instance, ROS production is considered to be an earlier step than A*β* deposition itself. ROS can disrupt cellular signaling by oxidizing lipids, proteins and DNA. Remarkably, this is a feature usually found in AD patients’ brains [30]. The combination of Fe/Asc was used to induce oxidative stress in our cellular model. As observed in Figure 4, Fe/Asc was able to decrease SH-SY5Y viability. In our assay, neither of the tested substances was able to protect cells against a Fe/Asc insult (Figure 4). These results might suggest that compounds **7**–**9** can provide neuroprotection independently of the oxidative status of cells.

## 3. Materials and Methods

### 3.1. Chemicals and Apparatus

For the synthesis of the compounds, all the reagents (from Sigma-Aldrich, St. Louis, MO, USA; Alfa-Aesar, Kandel, Germany; Acros, Thermo Fisher Scientific, Geel, Belgium) were of analytical grade and used as supplied. Whenever necessary, the solvents were dried according to standard methods [31]. The chemical reactions were followed by thin layer chromatography (TLC) using alumina plates coated with silica gel 60 F254 (Macherey-Nagel, Düren, Germany). Column chromatography purifications were performed on silica gel (Merck 230–400 mesh, Geduran Si 60, Darmstadt, Germany). The purity of the compounds was assessed by determination of the melting points (M.P.) and by using NMR and mass spectral techniques. The melting points were measured with a Leica Galen III (Microsystems, Wetzlar, Germany) hot stage apparatus and are uncorrected. ^1^H- and ^13^C-NMR spectra were recorded either on Bruker AVANCE III-300 (300 MHz and 75.5 MHz) or Bruker AVANCE III-400 (400 MHz and 100 MHz) NMR spectrometers (Billerica, MA, USA), at 25 °C. Chemical shifts are reported as δ values (ppm) from internal reference TMS (tetramethylsilane, Sigma-Aldrich, St. Louis, MO, USA) and coupling constants (*J*) in Hertz. The following abbreviations have been used: s = singlet, d = doublet, t = triplet, m = multiplet. The mass spectra were performed on a 500 MS LC Ion Trap mass spectrometer (Varian Inc., Palo Alto, CA, USA) equipped with an ESI ion source, operated in the positive ion mode.

### 3.2. Molecular Modeling: Docking and Pharmacokinetics Studies

Docking simulations were done using the Gold v. 5.1 software (CCDC, Cambridge, UK) [32]. The AChE active site model structure was retrieved from the protein database (RCSB) (PDB, entry 1 EVE) [33], as a complex of AChE with Donepezil (DNP), a clinical drug in use, herein named as the original ligand. Despite of some recognized differences between the inhibitor-enzyme complexes, described for the human acetylcholine esterase (*h*AChE) and the electric ray homologue (*Torpedo californica*, *Tc*AChE), both enzymes have shown to be conservative at the main amino acid residues that line the active site gorge [34] and so the docking study was performed using the *Tc*AChE.

For the docking simulations, the *Tc*AChE-DNP complex was retrieved from RCSB and then treated with MAESTRO v. 9.3 (Schrödinger Inc., Portland, OR, USA) [35], to obtain the receptor-enzyme model, by removing the original ligand, solvent and co-crystallization molecules and subsequent addition of hydrogen atoms. The program was also used to create the ligand configurations that were optimized (1000 cycles of random conformational search and 2500 optimization steps) using Ghemical v 2.0 (Free Software Foundation Inc., Atlanta, GA, USA) [36]. The ligands were then docked with the AChE active site, using GOLD program with default parameters and the Astex Statistical Potential (ASP) scoring function. The docking site was well-defined by selecting a box within 10 Å from the original ligand at the above stated PDB structure. The top structures generated from these computations were compared with the original ligand and their interactions evaluated with the active site residues. The outcomes were pictured using Chimera 1.6 (Free software, University of California, San Francisco, CA, USA) [37].

Pharmacokinetic molecular properties were assessed in silico. Factors like the ability to be absorbed through the intestinal tract (Caco-2 cell permeability), blood–brain barrier partition coefficient (log BB), the octanol–water partition coefficient (*c*log *P*) and CNS activity were estimated. The chemical structures were previously optimized using Maestro v. 9.3 (Schrödinger Inc., Portland, OR, USA) followed by submission in the calculation of these relevant pharmacokinetic properties and descriptors using QikProp v. 2.5 (Schrödinger, LLC, New York, NY, USA) [22].

### 3.3. Synthesis

#### 3.3.1. Synthesis of the Compounds **1** and **2**

Equivalent amounts of phthalic anhydride (1 eq) and of 1-(2-aminoethyl)-piperazine (**1a**) or (piperidin-4-yl)methanamine (**2a**) were heated at 160–180 °C for 4 h. The resulting dark brown solid was dissolved in ethanol 96% and subsequently were added equivalent amounts of KOH and benzyl bromide. The resulting mixture was stirred at room temperature (RT) for 24 h. Then, the ethanol was removed in vacuo and the residue was partitioned between diethyl ether and H_2_O. The aqueous layer was further extracted with diethyl ether (2 times) and the collected organic phases were washed with brine, dried over anhydrous Na_2_SO_4_, filtered and concentrated under reduced pressure. The crude was purified by chromatography column (eluent: DCM/MeOH 92:8 or ACN/H_2_O) and the pure products were obtained as pale-yellow solids (**1** or **2**).

*2-(2-(4-Benzylpiperazin-1-yl)ethyl)isoindoline-1,3-dione* (**1**). Yield 50%; ^1^H NMR (400 MHz, CDCl_3_), δ (ppm): 2.39–2.52 (m, 8H, piperazine), 2.60 (t, 2H, *J* = 6.7 Hz, phthalimide-CH_2_CH_2_N), 3.44 (s, 2H, NCH_2_Ph) 3.78 (t, 2H, *J* = 6.7 Hz, phthalimide–CH_2_CH_2_N), 7.19–7.27 (m, 5H, aromatics, Ph–CH_2_), 7.67–7.69 and 7.80–7.82 (m, 4H, phthalimide). *m*/*z* (ESI-MS): 350 (M + H)^+^.

*2-((1-Benzylpiperidin-4-yl)methyl)isoindoline-1,3-dione* (**2**). Yield: 29%; ^1^H NMR (300 MHz, DMSO-*d*_6_), δ (ppm): 1.07–1.15, 1.58–1.76 and 3.44–3.50 (m, 9H, piperidine), 2.73 (d, 2H, NCH_2_CH) 3.41 (s, 2H, NCH_2_Ph), 7.15–7.40 (m, 5H, aromatics, Ph–CH_2_), 7.79–7.91 (m, 4H, phthalimide). *m*/*z* (ESI-MS): 335 (M + H)^+^.

#### 3.3.2. Synthesis of the Compounds **3** and **4**

2-(2-(4-Benzylpiperazin-1-yl)ethyl)isoindoline-1,3-dione (**1**) or 2-((1-benzylpiperidin-4-yl)methyl)isoindoline-1,3-dione (**2**) (0.897 mmol) was dissolved in an aqueous solution of MeNH_2_ 40% (*w*/*w*, 5 mL) and stirred at room temperature for 60 h. Then, an aqueous solution of NaOH 20% (*w*/*w*, 5 mL) was added and the resulting mixture was stirred for 2 h. Afterwards NaCl was added (0.897 mmol) and the solution was extracted with dichloromethane and the organic layer washed with water, dried over anhydrous Na_2_SO_4_, filtered and concentrated under reduced pressure affording pale-yellow oils.

*2-(4-Benzylpiperazin-1-yl)ethanamine* (**3**). Yield: 97%. ^1^H NMR (400 MHz, CDCl_3_), δ (ppm): 2.35–2.43 (m, 10H, piperazine, NH_2_–CH_2_CH_2_N), 2.73 (t, 2H, *J* = 6.1 Hz, NH2–CH_2_CH_2_N), 3.46 (s, 2H, NCH_2_Ph), 7.18–7.27 (m, 5H, aromatics, Ph–CH_2_). M/z (ESI-MS): 220 (M + H)^+^.

*(1-Benzylpiperidin-4-yl)methanamine* (**4**). Yield: 69%. ^1^H NMR (300 MHz, DMSO-*d*_6_), δ(ppm): 1.00–1.23, 1.61–1.65, 1.82–1.89 and 2.37–2.39 (m, 9H, piperidine), 2,77 (d, 2H, *J* = 11.5 Hz, NCH_2_CH) 3.41 (s, 2H, NCH_2_Ph), 7.20–7.33 (m, 5H, aromatics, Ph–CH_2_). M/z (ESI-MS): 205 (M + H)^+^.

#### 3.3.3. Synthesis of the Final Conjugates (**5**–**9**)

To a solution of 2-(4-benzyl-1-piperazinyl)ethanamine (**1**) or (1-benzylpiperidin-4-yl)methanamine (**2**) (0.509 mmol) in dioxane (12,7 mL), was added the suitable sulfonyl chloride derivative (in a stoichiometric ratio 1:1.2) and stirred overnight at RT. The mixture was concentrated under reduced pressure, added to water and extracted with ethyl acetate for 3 times. The organic layers were washed with a solution of HCl 1M and NaOH 1M and then dried over anhydrous Na_2_SO_4_ and concentrated under reduced pressure. The crude was purified by chromatography column (eluent: DCM/MeOH, 95:5).

*N-(2-(4-benzylpiperazin-1-yl)ethyl)-4-methylbenzenesulfonamide* (**5**). Yield: 30%, yellow oil. ^1^H NMR (400 MHz, MeOD-*d*_4_) δ (ppm): 7.73 (d, *J* = 8.2 Hz, 2H: H21, H25), 7.38 (d, *J* = 8.0 Hz, 2H: H22, H24), 7.33–7.27 (m, 5H, H1-H6), 3.59 (s, 2H: H7), 2.97 (t, *J* = 6.7 Hz, 2H: H15), 2.70–2.44 (m, 10H, H9–H14), 2.42 (s, 3H, H26). ^13^C NMR (100 MHz, MeOD-*d*_4_) δ (ppm): 144.76, 138.71, 130.90, 130.76, 129.44, 128.77, 128.10, 63.57, 58.04, 53.37, 53.33, 40.91, 21.44. *m*/*z* (ESI-MS): 374.19 (M + H)^+^.

*N-(2-(4-benzylpiperazin-1-yl)ethyl)-4-methoxybenzenesulfonamide* (**6**). Yield: 49%, yellow oil. ^1^H NMR (300 MHz, MeOD-*d*_4_) δ (ppm): 7.78 (d, *J* = 8.9 Hz, 2H: H21, H25), 7.33–7.23 (m, 5H: H1-H6), 7.07 (d, *J* = 8.9 Hz, 2H: H22, H24), 3.87 (s, 3H: H27), 3.54 (s, 2H: H7), 2.96 (t, *J* = 6.8 Hz, 2H: H15), 2.56–2.41 (m, 10H: H9–H14). ^13^C NMR (75.5 MHz, MeOD-*d*_4_) δ(ppm): 165.45, 137.83, 133.13, 130.81, 130.20, 129.37, 128.60, 115.33, 63.73, 58.09, 56.21, 53.56, 53.47, 40.94. *m*/*z* (ESI-MS): 390.18 (M + H)^+^.

*N-(2-(4-benzylpiperazin-1yl)ethyl)-[1,1’-biphenyl]-4-sulfonamide* (**7**). Yield: 38%, white solid, M.P. 101-103 °C. ^1^H NMR (400 MHz, MeOD-*d*_4_) δ (ppm): 7.98–7.44 (m, 9H: H21-H31), 7.39–7.23 (m, 5H: H1-H6), 3.52 (s, 2H: H7), 3.05 (t, *J* = 6.3 Hz, 2H: H15), 2.68–2.22 (m, 10H: H9–H14). ^13^C NMR (75.5 MHz, MeOD-*d*_4_) δ(ppm): 146.67, 140.60, 140.40, 137.90, 130.78, 130.16, 129.53, 129.33, 128.67, 128.53, 128.28, 63.78, 58.16, 53.59, 53.51, 41.05. *m*/*z* (ESI-MS): 436.36 (M + H)^+^.

*N-((1-benzylpiperidin-4-yl)methyl)-4-methoxybenzenesulfonamide* (**8**). Yield: 39%, white solid. M.P. 83-86 °C. ^1^H NMR (400 MHz, MeOD-*d*_4_) δ (ppm): 7.78 (d, *J* = 7.9 Hz, 2H: H20, H24), 7.37–7.26 (m, 5H: H1–H6), 7.08 (d, *J* = 8.0 Hz, 2H: H21, H23), 3.89 (s, 3H: H26), 3.60 (s, 2H: H7), 2.95 (d, *J* = 11.5 Hz, 2H: H9′, H13′), 2.71 (d, *J* = 6.7 Hz, 2H: H14), 2.08 (dd, *J* = 19.3, 7.8 Hz, 2H: H10′, H12′), 1.72 (d, *J* = 12.7 Hz, 2H: H9′’ H13′’), 1.49–1.40 (m, 1H: H11), 1.27–1.18 (m, 2H: H10′’, H12′’). ^13^C NMR (75.5 MHz, MeOD-*d*_4_) δ(ppm): 133.41, 131.08, 130.07, 129.42, 128.81, 115.26, 63.86, 56.18, 54.02, 36.92, 30.05. *m*/*z* (ESI-MS): 375.17 (M + H)^+^.

*N-((1-benzylpiperidin-4-yl)methyl)-[1,1’-biphenyl]-4-sulfonamide* (**9**). Yield 48%, white solid. M.P. 139–140 °C. ^1^H NMR (400 MHz, MeOD) δ 7.91–7.39 (m, 9H: H20-H30), 7.30–7.20 (m, 5H: H1–H6), 3.51 (s, 2H: H7), 2.88 (d, *J* = 11.0 Hz, 2H: H9’,H13’), 2.76 (d, *J* = 6.5 Hz, 2H: H14), 2.04–1.96 (m, 2H: H10’,H12’), 1.70 (d, *J* = 12.9 Hz, 2H: H9’’,H13’’), 1.50–1.38 (m, 1H: H11), 1.29–1.16 (m, 2H: H10’’,H12’’). ^13^C NMR (75.5 MHz, MeOD-*d*_4_) δ (ppm): 146.59, 140.66, 140.64, 137.81, 130.95, 130.14, 129.49, 129.31, 128.63, 128.57, 128.28, 64.09, 54.11, 49.45, 37.16, 30.26. *m*/*z* (ESI-MS): 421.35 (M + H)^+^.

### 3.4. Biological Assays in Solution

#### 3.4.1. Acetylcholinesterase Inhibition

The acetylcholinesterase inhibitory activity was assessed by an adaptation of the Ellman’s method [38,39]. The assay solution contained 4-(2-hydroxyethyl)-1-piperazineethanesulfonic acid (HEPES) buffer (50 mM, pH 8.0), 5,5′-dithiobis-(2-nitrobenzoic acid) (DTNB, 3 mM), the stock solution of each compound in methanol (1 mg/mL), AChE (type VI-S, from electric eel) and the required quantity of methanol to reach the desired volume of sample mixture in a 1 mL cuvette. Samples were incubated for 15 min and then 75 μL of acetylthiocholine iodide (AThChI) solution (16 mM) was added. The reaction was monitored for 5 min at 405 nm. Assays were run with a blank containing all the reagents but AChE. Reaction rates were calculated as well as the enzyme activity. A positive control reaction was performed using methanol as a sample solution. The fraction of enzyme inhibition due to the existence of rising concentration of the test compound was calculated using Equation (1), where *v*i = starting reaction rate in the presence of inhibitor; *v*o = starting reaction rate of the control reaction.

(1) $$\%I\;=100-100\;\times \;\frac{vi}{\left(vi-vo\right)}$$

The inhibition curves were achieved by plotting the percentage of enzymatic inhibition against inhibitor concentration and a calibration curve was got from which the linear regression parameters were obtained.

#### 3.4.2. Inhibition of A*β*_1–42_ Aggregation

Inhibitory capacity of the compounds for the A*β*_1–42_ aggregation was assessed based on the fluorescence properties of thioflavine T (ThT), measured with a Varian Cary Eclipse spectrofluorometer.

A previously prepared solution of A*β*_1–42_ was used. Firstly. the lyophilized peptide (1 mg) was dissolved in 1,1,1,3,3,3-hexafluoro-2-propanol, (HFIP) (1.5 mL) by brief sonication and kept overnight at 25 °C. The following day, in an ice bath, the solution was partitioned into 6 Eppendorf tubes (250 μL) and left to evaporate overnight at 25 °C. Then, the resulting films were stored at −20 °C for months [14].

A film of A*β*_1–42_ was re-dissolved in a fresh mixture solution of CH_3_CN/Na_2_CO_3_ (300 μM)/NaOH (250 mM) (48.3/48.3/3.4 μL *v*/*v*/*v*) by brief sonication. To the resulting alkaline A*β*_1–42_ solution was added phosphate buffer (pH 8.0). The compounds tested were diluted in MeOH (1 mg/mL) and then further diluted in phosphate buffer. Two different types of experiments were prepared in a final volume of 60 μL. The solution for the control ligand assay consists in 30 µL of phosphate buffer and 30 µL of A*β*_1–42_ solution, to evaluate the aggregation without any inhibition, while the solution for the ligand assay consists of 20 µL of phosphate buffer, 30 µL of A*β*_1–42_ solution and 10 µL of the ligand solution, to evaluate the effect of the ligand in the aggregation. For each experiment a blank sample, without A*β*_1–42_, is used to monitor the effect of the compounds in the fluorescence. These samples were left incubating in a water bath (CERTOMAT WR), for 24 h, at 37 °C with gentle shaking [14].

A glycine-NaOH buffer (pH 8.5) containing ThT (5 μM) was added to each sample. Samples were placed in a microplate and the fluorescence signal was monitored at 446 nm (λ_exc_) and 490 nm (λ_em_) [40].

The percentage of inhibition was obtained, following the equation (2).

(2) $$\%I=100-\left(\frac{F_{i}}{F_{0}}\right)\times \;100$$

where F_i_ and F_0_ are the fluorescence intensities in the presence and absence of the compound, respectively, minus the fluorescence intensities of the respective blanks. Statistical analysis was performed in Microsoft Office Excel^®^.

### 3.5. Cell Viability and Neuroprotection

SH-SY5Y human neuroblastoma cells (ATCC-CRL-2266) (American Type Culture Collection, Manassas, Virgínia, USA) were plated in DMEM/F12 (Dulbecco’s Modified Eagle Medium/Ham’s F-12 Nutrient Mixture; Thermo Fisher Scientific) with 10% heat inactivated fetal bovine serum, 50 U/mL penicillin and 50 U/mL streptomycin under a humidified atmosphere with 95% relative humidity at 37 °C at a 0.16 × 10^6^ cells/mL density. Each of the tested substances (**6**, **7**, **8**, **9**) were dissolved in DMSO to prepare a stock solution at a 25 mM concentration and stored at −20 °C until further use. SH-SY5Y cells were treated for 25 h with varying concentrations of compounds, in order to select the highest non-toxic concentration. The selected concentrations for each compound were: 40 µM final concentration for **6**; 15 µM final concentration for **7**; 30 µM final concentration for **8** and 2.5 µM final concentration for **9**. The final concentration of DMSO did not exceed 0.05% (*v*/*v*) and no alterations on cells were observed. For the neuroprotection assays, the incubation with A*β*_1–42_ or L-ascorbic acid/ferrous sulphate treatment was preceded with 1 h incubation of the compounds when needed. Pre-incubation was followed by 24 h treatment of A*β*_1–42_ or Fe/Asc, accounting a total of 25 h. A*β*_1–42_ was prepared as 276.9 µM stock in sterile water and administrated in the medium at a 2.5 µM final concentration. Ferrous sulphate was freshly prepared as 0.36 M stock in water and added to the medium at 2.5 mM final concentration. L-ascorbic acid was freshly prepared as 80 mM stock in water and added to the medium at 5 mM final concentration. A*β*_1–42_ was purchased from Bachem (Torrance, CA, USA) and ferrous sulphate and L-ascorbic acid from Sigma Chemical Co. (St. Louis, MO, USA).

Cellular viability was determined by employing the MTT (3-(4,5-dimethylthiazol-2-yl)-2,5-diphenyltetrazolium bromide) reduction assay, according to the Mosmann method [41]. This colorimetric test consists in the ability of the succinate dehydrogenase enzyme from viable cells to metabolize the MTT into a formazan. In the end of treatments, cells medium was aspirated and cells were washed with 1 × PBS (phosphate-buffered saline) to remove dead cells. MTT solution (200 µL, 0.5 mg/mL) was added to each well and incubated for 2 h protected from light. Formazan precipitates were then dissolved in DMSO and absorbance measured at 570 nm. The ability to reduce MTT was expressed as a percentage of untreated, control cells.

## 4. Conclusions

AD is recognized as a multifactorial complex disorder but current approved therapeutics, such as the acetylcholinesterase (AChE) inhibitor donepezil (DNP), only provides symptomatic relief. Following the promising recently adopted strategy of single molecule-multiple target drugs associated with the advantage of repositioning already approved drugs, a new set of DNP-sulfonamide hybrids was herein developed. These compounds include the DNP pharmacophore nucleus (*N*-benzylpiperidine or its analogue *N*-benzylpiperazine) connected to substituted arylsulfonamide derivatives aimed to improve the binding with AChE and its inhibition, as well as to inhibit the formation of amyloid-beta (A*β*) aggregates. The designed series of hybrids was synthesized, fully characterized and bioassayed. The results of in vitro assays indicated a good inhibitory potential against AChE and A*β*_1–42_ aggregation, namely for the benzylpiperidine-aryl sulfonamide derivatives (**8** and **9)**, but in particular **9** (AChE (IC_50_ = 1.6 μM) and 60.7% A*β*_1–42_ aggregation), substantiating the computational simulation studies. Some compounds evidenced neuroprotection of SHSY5Y neuroblastoma cells, namely reverting neurotoxicity induced by A*β* peptides. Importantly, the compounds also show good drug-like properties with potential oral formulation. Overall, the results indicate compound **9** as the most promising DNP-sulfonamide hybrid of this series, which can be considered as a lead compound for further developments on anti-AD drugs.

## Figures and Tables

**Table 1:** Summary of pharmacokinetic properties predicted by the software QikProp v.2.5.

Compound	MW(Da)	*c*log *P*	log BB	Caco Permeability (nm/s)	CNS
**5**	373.512	2.259	0.123	114	+
**6**	389.512	1.972	0.094	116	+
**7**	435.583	3.575	0.021	118	+
**8**	374.497	3.080	−0.354	363	+
**9**	420.568	4.686	−0.319	457	+
**DNP**	379.50	4.269	0.132	893	+

**Table 2:** Biological activity of the compounds towards the inhibition of AChE and A*β*_1–42_ aggregation.

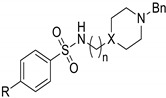	X	R	n	AChE Inhib.^a^ IC_50_ (μM) ± SD	A*β*_1–42_ Aggreg. Inhib.^b,c^ (%)
5	N	Me	2	≥100	41.8
6	N	OMe	2	89 ± 9	37.2
7	N	Ph	2	38.7 ± 0.2	34.6
8	CH	OMe	1	6.2 ± 0.9	64.2
9	CH	Ph	1	1.6 ± 0.2	60.7
Tacrine	−	−	−	0.15 ± 0.02	54.5
DNP ^d^	−	−	−	0.026	<5

^a^ The values are mean of two independent experiments ± SD; AChE from *Electric eel*; Tacrine was used as a control; ^b^ Inhibition of self-induced A*β*_1–42_ aggregation (%) by the thioflavin-T fluorescence method in the presence of the inhibitor (40 μM); Tacrine was used as a control; ^c^ The values are the mean of three independent measurements in duplicate (SEM < 10%); ^d^ references [3,27].

## Data Availability

The data presented in this study is available in the article.

## Sample Availability

Samples of the compounds are not available from the authors
